# Development of evidence-informed bridge programming to support an increased need for eating disorder services during the COVID-19 pandemic

**DOI:** 10.1186/s40337-022-00590-1

**Published:** 2022-05-20

**Authors:** Lindsey D. Bruett, Sarah Forsberg, Erin C. Accurso, Sasha Gorrell, Lisa Hail, Jessica Keyser, Daniel Le Grange, Kathryn M. Huryk

**Affiliations:** 1 Department of Psychiatry and Behavioral Sciences, UCSF Weill Institute for Neurosciences, University of California, San Francisco, 401 Parnassus Ave., San Francisco, CA 94143 USA; 2 Department of Psychiatry and Behavioral Neuroscience, The University of Chicago, Chicago, IL USA

**Keywords:** Eating disorder, Anorexia nervosa, Atypical anorexia nervosa, Children and adolescents, Young adults, Group therapy, Brief intervention, Family-based treatment, Treatment access

## Abstract

Over the course of the COVID-19 pandemic, rates of eating disorders have increased, further straining systems of care that were already overburdened. The current paper describes novel interventions, largely informed by Family-Based Treatment (FBT), that were implemented by a tertiary specialist adolescent eating disorders service. In response to the pandemic, programming was designed to bridge access to care while waiting for availability of evidence-based therapy. The Brief Psychology Consultation Clinic provides several sessions to patients and families, focused on psychoeducation and problem-solving informed by FBT and other evidence-based therapies. Two groups, the FBT Caregiver Workshop Series and FBT Caregiver Support Group, provide psychoeducation and support for caregivers of youth with eating disorders. Perceived strengths and benefits of these services, as well as barriers to implementation and future research directions are discussed.

## Introduction

The COVID-19 pandemic has been associated with a perceived increase in the incidence and severity of eating disorders (EDs) [1]. While still largely anecdotal, the pandemic appears to have presented a range of non-specific risk factors and stressors increasing vulnerability to the development of EDs. For example, the disruption of daily routine and lack of regular structure, as well as decreased food availability (e.g., navigating food scarcity in the beginning stages of mandated lockdown) have been identified as vulnerabilities [2, 3].

**Fig. 1:**
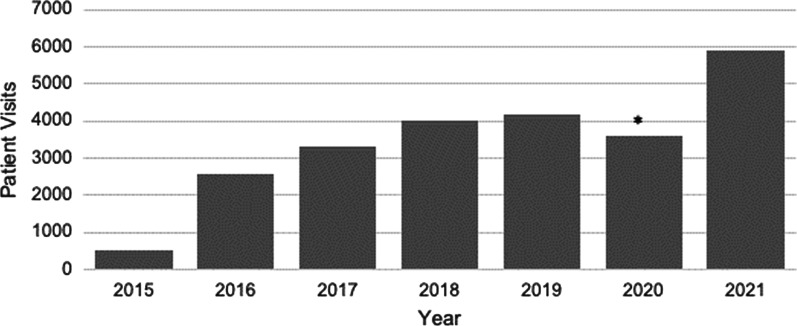
Seven Year Growth in Patient Visits at UCSF Eating Disorders Program. *Note* Outpatient behavioral health and medical visits at UCSF Eating Disorders Program, 2015–2021. *Reflects temporary reduction in clinical service when the World Health Organization declared COVID-19 a pandemic. We also were not capturing video visits early in the pandemic

The apparent exacerbation of EDs during the pandemic has collided with existing constraints on access to specialized treatment [4]. Subsequently, there have been reported increases in the number of ED-related medical hospitalizations in several parts of the world, consequently straining systems of care [4–6]. For instance, chart reviews at a pediatric hospital in the upper midwestern United States evidenced a consistent rate of admissions prior to the pandemic with a brief dip in April 2020 followed by an increase of more than double (123%) between April 2020 and March 2021 [7]. These increases are consistent with our own Eating Disorders Program (EDP) at the University of California, San Francisco (UCSF), where we have seen a more than doubling of the typical medical inpatient census, and significant increase in outpatient visits in 2021 (see Fig. 1).

Increases in ED prevalence rates have put pressure on already-stretched systems of care to meet the needs of the volume of patients in certain catchment areas, e.g. New Zealand [5]. Historical review of studies of community samples report that even prior to the COVID-19 pandemic, less than half of individuals with an ED gained access to care despite the majority of individuals expressing a desire for treatment [8]. An early-pandemic survey of over 1000 individuals in the United States and the Netherlands revealed that only about half of those surveyed were receiving ED treatment [3]. These gaps in care are exacerbated for marginalized groups, who face additional systemic inequities and barriers to ED treatment [9, 10].

Family-based treatment (FBT), the leading treatment for adolescent restrictive spectrum EDs (e.g., Anorexia Nervosa (AN), Atypical Anorexia Nervosa (AAN), many presentations of Other Specified Feeding and Eating Disorder (OSFED)) [11], can be even harder for families to access than less specialized care given that, within the United States, only 52 providers within 13 states have completed the FBT certification process [12]. This leaves many youth with EDs with limited access to specialized treatment. Prior to the pandemic, our program typically operated with a waitlist of about 2 months before initial psychological evaluation, followed by a 2–3 month wait to start of therapy. During the pandemic, wait times have increased significantly, now averaging 2–4 months for an initial psychological evaluation, followed by a 3–6 month wait before starting therapy. The difference in demand for services is actually much greater, but the majority of patients on the waitlist ultimately connect with a community-based provider and decide not to transfer services to UCSF several months after starting treatment with another provider, most commonly using a different treatment model than what would be offered within our program. Further, and again anecdotally, colleagues who provide evidence-based treatment for EDs in private practice settings in the state of California have been reporting similarly full practices and long waits for care. Likely due to the widespread adoption of telehealth since the start of the pandemic [13], our referrals have also increased in geographical spread (e.g., from other regions in the state of California), allowing for greater access to specialized treatment but creating additional strain on our existing system of care.

EDs are among the deadliest of the psychiatric disorders [14], and negatively impact not only the physical health and quality of life of patients but also increase disruptions and decrease quality of life for their families [15]. Many EDs develop during adolescence and can become severe and enduring in course, with the majority of individuals with EDs remaining undiagnosed and untreated [8]. Prognosis is more favorable with earlier diagnosis and treatment [16–18] as treatment may become less effective with increased duration of illness [19]. As such, prompt access to high quality care for individuals with EDs is of utmost importance.

Below, we describe efforts at the UCSF EDP to respond to pandemic-era needs and provide accessible care to a wider range of patients and families impacted by EDs.

## Description of UCSF Eating Disorders Program

The UCSF EDP is a comprehensive multidisciplinary program that provides clinical care for individuals with EDs up to age 25 and their families through a collaboration between the Department of Psychiatry and Behavioral Sciences and the Department of Pediatrics. Services include comprehensive psychiatric, medical and nutrition evaluations, followed by evidence-based individual and family therapies, ongoing medical management (including inpatient medical stabilization), nutrition counseling, social work support, and medication management. Most youth treated within the program are adolescents who meet criteria for restrictive spectrum EDs (e.g., AN, AAN, OSFED). In keeping with the FBT model and its evidence base for these disorders [20], and also to maximize patient access across our evidence-based services, outpatient psychotherapy is largely informed by FBT principles and intended to be short-term (e.g., 6–12 months). Over the past three years, our program also developed a five-day intensive program for patients with restrictive-spectrum EDs and their families, adapted from existing Multi-Family Group Treatment models [21, 22] in order to expand access to FBT-informed care beyond Northern California. However, we suspended this programming in March 2020 due to the onset of the COVID-19 pandemic. Our programming could not be easily adapted to a virtual multifamily format, and it was determined that a single-family format was not an effective use of limited resources given disruptions to ongoing clinical care and long waitlists to enter outpatient mental health care.

## Novel bridge programming in response to the COVID-19 pandemic

With the onset of the pandemic, our focus shifted to identifying strategies to improve access to care, as it became clear that community resources were also overwhelmed with referrals, with patients and their families waiting several months to receive treatment. Our clinical service has been inundated with new patients and former patients experiencing return of symptoms since the summer of 2020 (Fig. 1), driven in part by increased referrals from primary care (general practice) providers, and also the more than two-fold increase in ED-related inpatient medical admissions on our service [23].

We describe new programming below, which centers around our collective prioritization of delivering brief interventions that can help families learn and apply principles of evidence-based treatment, with low barriers to entry. These services, collectively referred to as bridge programming, were designed to address the gap between establishment of medical services (inpatient and/or outpatient) and initiation of mental health treatment within our team or with other appropriate community providers. At this time, our bridge programming is provided exclusively via telehealth, which has an added benefit of serving a wider catchment area. The data we present are exempt from human subjects review by our Institutional Review Board as they represent quality improvement efforts and are not considered human subjects research. Below, we present some preliminary outcomes resulting from our development of bridge programming.

## Bridge programming overview

### Behavioral health consultation in medical settings

The provision of behavioral health consultation within medical settings (e.g., pediatrics) is a documented strategy aimed at promoting access to behavioral health care, serving a broader range of patients, and preventing behavioral health problems [24, 25]. In this model, the behavioral health consultant acts as a primary member of the care team, which has been demonstrated to increase access to behavioral health services, particularly for patients who may not follow through with a physician referral to an outside behavioral health provider [26]. The behavioral health consultant typically meets with patients and families in clinic at the time of a medical visit, providing education and brief, focused problem-solving. Documentation occurs in the same chart and providers use a shared treatment plan [24]. Building on aspects of behavioral health consultation in primary care settings, we developed the Brief Psychology Consultation Clinic (BPCC), described below. Our model differs from many primary care consultation models in two important ways: (1) while we aim to prevent worsening of symptoms, most patients referred already meet criteria for a diagnosable behavioral health condition, and (2) the BPCC consultation visit is not scheduled in tandem with a medical visit.

### Brief Psychology Consultation Clinic

#### Overview

The BPCC is a clinical service that offers up to three 30-min consultation sessions with a psychologist with expertise in EDs and general child, adolescent, and young adult mental health. BPCC supports patients and families already seen by the UCSF Adolescent and Young Adult Medicine arm of the EDP, and who are (1) awaiting ED-specific behavioral health intervention, or (2) without an ED but with other mental health concerns or stress. Patients who were hospitalized are eligible for an additional consultation post-discharge.

#### Referrals

Referrals are generated by team providers (physicians, dieticians, social workers, inpatient psychologists, or outpatient psychologists completing new patient evaluations). Prior to the visit, the BPCC psychologist reviews the chart and referral targets (provided by referring clinician).

#### Logistics

Referring providers discuss potential referrals with patients and/or their families, clarifying that it is not a substitute for longer-term therapy. With family consent, the provider submits a brief referral to clinic administrative staff and the BPCC psychologist. The referral includes targets for the visit and a recommendation for who should attend (e.g., patient, caregivers, both). Administrative staff assist with checking mental health insurance benefits and scheduling the first consult. The BPCC psychologist meets with the family via telehealth and may schedule up to two follow-up visits when appropriate, generally spaced one month apart. However, time to follow-up can vary based on clinical need and appointment availability. BPCC visits are billed using the appropriate individual or family therapy code.

#### Content

At the first visit, a brief overview of the BPCC, confidentiality, and limits to confidentiality are provided. Session content is tailored to the patient’s/family’s needs and questions and may include psychoeducation about mental health topics and/or treatment (e.g., orientation to FBT) or targeted behavioral strategies to address presenting concerns (e.g., parental management of ED behaviors, behavioral activation for depression, sleep hygiene techniques) (see Table 1 for an expanded description of referral targets).

**Table 1:** BPCC referral targets

Appropriate referral targets for BPCC	
ED-related:	Non ED-related, general mental health concerns:
Basic education about: EDs, FBT principles, principles of managing ARFID-like symptoms (e.g., increasing flexibility)	Supporting caregivers who endorse challenges managing youth behavior (e.g., difficulty setting limits or reinforcing appropriate behaviors)
Reducing caregiver blame of self or child	Behavioral sleep techniques
Helping caregiver differentiate between ED driven behaviors/emotional response and other challenges	Helping patient determine a coping plan or learn strategies for managing mental health challenges (e.g., general and COVID-related stress, anxiety, depression)
Increasing caregiver effectiveness during meal/snack supervision	
Understanding the psychological components of exercise related to the ED	
Planning for college or other transitions	
For young adults living independently, tips to improve regular eating/decrease ED behaviors	

Referrals not appropriate for BPCC	
Target	Recommended support
Safety planning	Clinicians address as usual in session, per clinic protocol
In-depth diagnostic or treatment planning questions	Refer to a community therapist, or for comprehensive mental health evaluation with our team
Need to connect to care and having trouble finding a therapist and/or not reaching out to schedule	Provide family with referral lists as usual
Advocacy for mental health services with family's health insurance plan	Addressed by social work
School advocacy work including 504 plan needs	Addressed by social work

#### Implementation

At the time of writing, BPCC has been offered for 19 months, receiving 268 unique patient referrals during that time, with an average of 16 BPCC referrals per month in 2021. A majority (69.8%) of referrals were made by medical practitioners, 18.7% by psychologists, and 11.6% by social workers. While referrals are open to any patient served by our program as outlined above, only a small minority of referrals (~ 1%) have been for patients without concern for an ED. This underscores the overwhelming need for ED-specific support in the Adolescent and Young Adult Medicine patient population.

On average, families waited about one month (*M* = 33 days, *SD* = 24.22, range: [0,153]) from the date of referral to attending their first BPCC session. Of note, administrative demands (see below) have resulted in longer wait times for some patients/families.

Of the 268 referrals, 55% have completed at least one BPCC visit at the time of writing (1 visit, *n* = 88; 2 visits, *n* = 35; 3 visits, *n* = 21; 4 visits, *n* = 3). A few patients were offered an additional session due to medical provider request and/or demonstrated ability to make progress in BPCC (e.g., expanding foods through monthly visits in the case of ARFID) while still proactively seeking long-term therapy. About two-fifths (*n* = 106, 40%) of patients referred more than one month ago never scheduled or attended a visit. Once scheduled, some reasons for not attending include connecting with evidence-based therapy before the first BPCC session, deciding not to attend based on high co-pays or insurance deductible, or not responding to scheduling outreach efforts. Fifteen patients referred within the past month have not yet had a visit scheduled, largely due to administrative delays rather than clinical capacity.

Of patients who completed one or two BPCC visits, 62% were invited to schedule an additional visit at their discretion but have yet to do so, and 10% have a future visit scheduled at the time of writing. For the remaining 28%, it was determined that BPCC follow-up was not appropriate, typically due to the family connecting with ongoing evidence-based therapy. Note that for the above outcomes, 16 additional patients were referred but had missing data from chart review and were excluded from all counts.

#### Perceived benefits/strengths

Anecdotally, families have expressed gratitude for the opportunity to seek consultation from a psychologist while they await connection to longer-term therapy. For families who have educated themselves about ED management, they often specifically benefit from problem-solving to tailor strategies to their child, and/or from endorsement that the strategies they have identified are appropriate given their child’s presenting concerns. For families who are more hesitant to intervene on apparent ED and/or other mental health concerns, we have found that education about the importance of early intervention from a BPCC psychologist can be helpful in reinforcing medical provider recommendations about connecting to appropriate care (i.e., pursuing mental health referrals provided).

The BPCC increases access to mental health care, by (1) providing services to families via telehealth appointments scheduled one at a time, rather than requiring families to commit to weekly visits and (2) ensuring significantly more families are served by our integrated service. It also reduces the burden on our UCSF mental health services by providing brief, targeted treatment for mild mental health symptoms which may not require long-term treatment, potentially reducing the length of treatment by providing skills to families while they await therapy, and providing more tailored referrals for families who may not be appropriate for care within our program.

The BPCC increases efficiency of patient visits for our non-psychologist colleagues (e.g., medical practitioners, dieticians) on our integrated care team who are otherwise taxed with providing mental health support (outside of their areas of expertise) to families while they await care. Following an initial two-month pilot of the BPCC, 100% (*N* = 16) of referring providers surveyed recommended continuing the service. Referring provider qualitative feedback at that time was positive and included several broad themes: helping families access necessary care more urgently, sending an important message to families to get started on nutritional rehabilitation at home, and allowing each provider to focus on their own scope of care rather than stretching to respond to mental health-related concerns. Additionally, the BPCC provides a rich training experience for learners in our multi-disciplinary team who may not otherwise have access to observing FBT principles in action.

#### Challenges and barriers to implementation

The largest challenge to date with implementing the BPCC service has been the burden on our limited administrative staff, who are responsible for (1) contacting each family’s insurance company to obtain benefits information, (2) calling families to inform them of benefits (e.g., amount of copay and/or deductible) and to schedule their first BPCC session, and (3) updating referring providers about the status of the referral. This process is time-consuming and difficult to sustain, at times resulting in available consultation slots going unscheduled despite a large number of unserved referrals.

Given that the BPCC psychologist has finite clinical time, providing BPCC services reduces available time to provide other outpatient mental health services (i.e., evaluation, longer term therapy). However, given that BPCC sessions are shorter than a typical therapy session (30 vs. 45–60 min), more families can be seen in a given clinic day. Further, many more families are served by BPCC than longer term therapy given the lower number of visits provided (1–3 visits vs. 20 + visits in a typical course of FBT).

Given that session content is responsive to the needs of the family (and therefore not always predictable ahead of session), and is delivered in a limited amount of time, the current BPCC model requires that the consulting clinician has considerable expertise in ED treatment and general child, adolescent, and young adult mental health concerns. Further, it requires that this clinician have familiarity with relevant resources for families (e.g., referrals, educational materials).

### Group services

Over the past decade, there has been growing interest and preliminary data to support “dual-generation” interventions for pediatric psychiatric disorders [27] whereby parent-only training, typically in a group format, is employed to enable caregivers to more effectively support their child. Among youth with EDs, several parent-targeted interventions have been examined, including parent-to-parent coaching as an augmentation of standard FBT [28], multi-family group formats of FBT for adolescent AN (MFT) [21, 29, 30], and an internet-based parent-only chat support group for parents participating in FBT [31]. MFT groups and caregiver-only groups have been implemented primarily in higher level of care settings alongside several other interventions, making it difficult to discern whether their addition impacts treatment outcome [32, 33]. Benefits of outpatient MFT interventions may include reductions in caregiver burden and parental isolation [30] as well as greater improvements in ED symptoms compared to those receiving family therapy alone [32]. Other emerging evidence for brief parent psychoeducational skills groups suggest they may improve early weight gain, parental knowledge, and confidence, above and beyond improvements made in FBT alone [34], and may be used to promote improvements in weight and ED psychopathology at initial presentation in the absence of other psychotherapy [35, 36]. Similar improvements in patient outcomes are observed with varied methods of providing parent education (e.g., workshop setting, online, guided-self-help modules) [37], and parents may additionally benefit from contact with other families [38]. Further, data on a parent-focused form of FBT supports the notion that caregivers can promote ED recovery in youth, even when youth involvement in treatment is limited [39]. Building on these data, we developed two group treatments for caregivers, described in detail below.

### FBT Caregiver Workshop Series

#### Overview

The FBT Caregiver Workshop Series is a four-session series of multi-family skill-building workshops created at the UCSF EDP for caregivers who want to learn more about implementing FBT. Group leaders orient caregivers to the principles of FBT and provide teaching on relevant topics, including nutrition, family communication, parenting strategies, and stress management. Facilitated group discussions among caregivers aim to enhance learning and create a supportive community. This series of workshops was designed for families who were not yet able to access specialty ED treatment, as well as those at the start or middle of FBT who want to bolster their work and connect with other caregivers. The FBT Caregiver Workshop Series is specified as not intended to be a substitute for FBT or other forms of psychotherapy.

This group was designed to meet via telehealth, once per week for 90 min across four consecutive weeks. We typically aim to enroll between 7 and 10 families at a time, or approximately 8–16 individual caregivers per group cycle. This is a closed group, meaning each individual caregiver is asked to commit to attending all four meetings in a cycle. Groups are led by 1–2 facilitators, comprised of a psychologist who is well-versed in FBT and occasionally also an advanced clinical psychology predoctoral intern or child and adolescent psychiatry fellow. Each workshop involves a combination of brief didactics and facilitated group discussions, described below.

#### Referrals

Referrals are generated from within the UCSF EDP, wherein all identified patients receive ongoing medical monitoring with our program adolescent medicine providers. Group participants are parents or other involved caregivers (e.g., grandparents, step-parents) of youth with an ED for whom outpatient FBT is indicated, primarily caregivers of those with AN or AAN but also other EDs involving marked dietary restriction (e.g., bulimia nervosa and other specified presentations). Some caregivers have begun or continued in the group while their children were in higher levels of care, including inpatient medical hospitalization. For safety, all identified patients must have completed some form of assessment with a UCSF EDP psychologist, such as a brief screening, BPCC, or a diagnostic evaluation. Suicidality and non-suicidal self-injury are assessed and managed in that context, and outpatients with significant safety concerns are not suitable for the group unless there is a plan for risk management with another provider.

#### Logistics

Administrative staff field internal referrals and register families for the group. Before the first meeting, the group leader sends a message via the electronic medical record system welcoming caregivers to the group, providing telehealth guidelines, and setting a tone for empowerment and support. The group is billed through insurance as multi-family group therapy.

#### Content and delivery

Each of the four workshops in this series focuses on topics and skills relevant to FBT. The group materials were informed by the FBT treatment manual [40], as well as programming developed by the UCSF EDP team for our ED-IFT program described above. The first workshop focuses on orienting caregivers to the group, including a review of confidentiality and group telehealth consent. Topics for teaching and discussion in the first group include: the core principles and phases of FBT, the consequences of starvation, externalization of illness, and barriers to independent recovery. The second workshop covers a topics related to mealtime management in FBT, such as nutrition and communication, with the aim of helping parents identify strategies they may use to effectively re-nourish their child. The third workshop focuses on monitoring progress and improving communication with validation. In the final workshop, caregivers discuss stress management and self-compassion. Caregivers are invited to reflect on what they’ve learned, provide feedback on the group, and share goodbyes.

In the style of FBT, facilitators aim to elicit information and advice from the caregivers themselves whenever possible. The facilitators review the workshop guidelines at the outset of each meeting, including reminders to defer to their primary treatment team for specific recommendations and that there is no “one-size-fits-all” approach to treatment. We also review the assumptions of FBT, including the belief that parents and families do not cause EDs, and are the most valuable agents of change in ED recovery. To engage parents and promote skills generalization, parents are presented with an assignment at the end of each meeting to be discussed in the following group. Facilitators also send a follow-up message after each workshop with supplementary resources**.**

#### Implementation

At the time of this writing, we have conducted eight rounds of the 4-week FBT Caregiver Workshop Series. From approximately 100 internally referred families who have expressed interest in the group, a total of 66 families (110 caregivers) have participated.

#### Perceived benefits/strengths

Anecdotally, clinicians in our program have observed how several families made significant progress with re-nourishment while participating in the group as a sole behavioral health intervention alongside ongoing medical monitoring. Others noted that when families participated in the group, they were more prepared to “dive in” when they eventually began formal FBT sessions with a therapist, and several adolescents were nearly weight-restored such that the targets for treatment were those generally addressed in later stages of FBT. These providers noted that because families had already received psychoeducation about FBT and were engaged in the treatment model, they were able to devote more time in-session to refining their efforts.

Caregivers have shared overwhelmingly positive feedback about the group. Many have noted feeling as if they better understand their child’s ED and how to help them recover after participating in this series of workshops. Caregivers appeared to absorb the principles of FBT in this group format, anecdotally describing increased focus on reversing the crisis of malnutrition and reduced concern with searching for possible causes of the ED. Several parents observed that they were better able to separate the ED from their child after hearing other caregivers describe similar behaviors in their children. Likewise, some shared that they felt less blame for causing the ED after connecting with other compassionate parents. Although most families had just learned of their child’s ED diagnosis, others were several months into implementing FBT strategies and were able to offer hope and credible advice. Those already working with an FBT provider described how discussing EDs and FBT with other families enhanced their learning and sense of empowerment. Caregivers have often noted that the group helped to reduce feelings of isolation and shame by connecting with other families who face similar challenges. In the words of one parent, *“It was good to hear how everyone’s struggle is similar to ours. We aren’t alone.”*

#### Challenges and barriers to implementation

Beyond the startup costs of initiating the referral process and creating group materials, this series of workshops is relatively low resource to implement, given its standardized and cyclical structure. This helps to ensure that leading the group has a negligible impact on the service’s capacity to deliver standard FBT. We have spaced out each series by 1–2 weeks to allow time for recruiting new participants and preparing incoming co-facilitators. The greatest challenge has been maintaining caregiver participation. Most caregivers who registered have completed the whole 4-week series, although attendance has varied across families and tended to be lowest in the fourth (final) workshop. Barriers to consistent attendance and engagement in the group have included competing responsibilities (e.g., work, caregiving) and visits with other treatment providers. Further, some insurance plans do not allow for an FBT and group session to occur on the same day, contributing to scheduling challenges. Over the past nine months, 7 families (10.6%) formally withdrew from the group following the first or second session, most often due to scheduling conflicts, time constraints, or changes in their treatment plan.

Although this was rare, a few parents reported feeling emotionally overwhelmed by listening to other families share their experiences with EDs. In the early sessions of standard FBT, the therapist does typically aim to raise parental anxiety, but only to the extent that it will motivate an adaptive response. One downside of the group format is that the therapist cannot as readily tune in to each caregivers’ individual level of distress, nor tailor the content and delivery to promote the optimal level of activation for each parent. Similarly, the group leader may not be able to target other important issues, such as family communication and expressed emotion, with the level of nuance and responsiveness we aim for in a standard family session. Caregivers could become skeptical of the FBT model or quickly burned out when the group intervention is not adequate nor flexible enough to meet their unique needs. Some may also feel that their child’s psychological needs are not being met if they begin implementing FBT without the support of a therapist who meets with the patient individually, particularly if safety concerns emerge.

### FBT Caregiver Support Group

#### Overview

The FBT Caregiver Support Group is an ongoing, open group created specifically for caregivers who have graduated from the FBT Caregiver Workshop Series as well as families already established in FBT at UCSF seeking additional support. The group was created following the completion of the first FBT Caregiver Workshop Series to address a need that caregivers expressed—an interest in continuing to meet with one another for support in implementing FBT principles at home. Caregivers’ qualitative feedback indicated a strong desire to maintain connection to other families with shared lived experience, reporting that their participation in group mitigated feelings of isolation and emotional strain in caring for their children. The group was designed to mirror these needs, aiming to facilitate connection and decrease caregiver strain while implementing FBT. Further, the group is hypothesized to bolster caregiver confidence in FBT, as caregivers provide encouragement and share practical strategies. Should the group achieve these aims, it has the potential to increase parental self-efficacy, an identified early predictor of success in FBT [41].

#### Referrals

Referrals for the group are placed by the treating clinician (Caregiver Workshop group leaders or FBT therapist) for caregivers of a child with AN, AAN, or OSFED.

#### Logistics

A psychologist with expertise in FBT leads the group, which is held every other week for one hour via telehealth and billed through insurance as multi-family group therapy.

#### Content

Group aims are largely to (1) reinforce FBT principles through transfer of learning between families and (2) decrease the sense of isolation that often comes with caring for a loved one with an ED. The group facilitator elicits problem-solving, feedback, and emotional support from caregivers themselves to foster a sense of empowerment and highlight shared experiences. The focus is on amplifying parental wisdom to increase a sense of efficacy, while providing psychoeducation and feedback (as needed) to highlight FBT concepts. As such, the structure involves a review of group guidelines, caregiver introductions (which include a brief summary of the family’s experience in treatment, to date)**,** and eliciting questions/topic areas for discussion.

Topics range from problem-solving strategies intended to support mealtime distress, navigating a return to school and/or athletics/physical activity, transitioning between FBT phases, managing roadblocks and plateaus, exploring definitions of recovery, and addressing caregiver distress and burnout associated with the experience of caring for an ill child. The primary interventions employed by the group facilitator are active listening, validation, reinforcement of positive changes, offering solutions generated by other families, highlighting FBT principles, and providing psychoeducation. The FBT assumptions described above in the FBT Caregiver Workshop Series are highlighted, and caregivers are reminded to work with their primary treatment team for specific recommendations.

#### Implementation

To date, 34 families have expressed interest in joining the group, of whom 20 families (23 caregivers) have attended at least one group. The group is open, and as such, families do not have to commit to attending all groups. As a result, group member participation has varied week to week, with some caregivers electing to attend regularly and others attending more variably. The number of participants has ranged from 2 to 7 per session. All caregivers participating in the group are connected to a mental health provider through either a UCSF EDP psychologist providing FBT, a provider in the community (FBT and other models), or a higher-level-of-care setting. The families participating in higher levels of care stated that they elected to attend group to prepare for their child’s transition to outpatient therapy, and to receive emotional support. As such, participating families are in all stages of treatment. Although some families are engaged in treatment other than formal FBT, all participating families value and are implementing principles of FBT at home with their child.

#### Perceived benefits/strengths

The families who attend group have frequently commented that having a space to meet with others who understand what they are going through is one of the most helpful aspects of group. Many highlight that family and friends are well-meaning and try to be supportive but cannot truly understand the significant stress and emotional strain of caring for a child with an ED. Families have regularly shared specific suggestions to address common challenges in FBT (e.g., navigating mealtime distress, managing parental emotions, nutrition strategies). While participating families are often in different phases of treatment, they describe a sense of emotional connection and feeling of being understood without having to explain themselves. Hearing one another’s struggles and how they have overcome them or learned to cope more effectively may present an opening for self-reflection and perspective-taking that otherwise may be more difficult when experienced in isolation or in traditional therapy. Caregivers genuinely celebrate each other’s progress, which can offer hope to those who are struggling or in earlier stages of treatment.

#### Challenges and barriers to implementation

Thus far, the primary barrier to implementation involves scheduling families. The group configuration can vary from week to week, and as noted, families are not required to attend all groups. This may explain a high rate of group no-shows (i.e., signing up earlier and forgetting to attend, having other commitments related to work or other treatment appointments that interfere). Our health care system does not have financial penalties for no-showed sessions. As noted above, some insurance plans do not permit an FBT and group session to occur on the same day, further complicating scheduling.

As with above services, provision of the group reduces the capacity of the clinician to provide other outpatient services. However, the impact is minimal (0.5 therapy slots per week).

## Conclusions and future directions

In 2020, the COVID-19 pandemic placed increased stress on our local system of care, which had already been struggling to meet the treatment demand of the communities we serve. This created an urgent need for the development of innovative approaches to treatment delivery, designed to decrease wait-times and improve access to care for patients and families, without exacerbating burden on our already limited resources. Rooted in evidence-based approaches to the treatment of EDs in youth, the BPCC and FBT Caregiver Workshop Series and Support Groups have allowed us to extend the reach and availability of our services with relatively low additional investment of time, effort, and expense. These bridge programming services have provided evidence-informed support when families would have otherwise been without care beyond medical monitoring, due to a burdened mental health care system lacking adequate capacity to meet community needs. Development of our bridge programming was made possible, in large part, by the rapid scale-up of telehealth during the pandemic. Providing these services in-person may not have been as successful. Indeed, we previously experienced difficulty with retention for in-person caregiver support groups, as families from a large catchment area were largely unlikely to travel to attend our in-person services in between work and caregiving responsibilities.

While our bridge programming was initially created out of an urgent need generated by the pandemic to fill seemingly unmanageable gaps in care, these interventions may continue to fill a gap given high demand for clinical services even before the pandemic. Indeed, waitlists of 4 to 6 months for therapy were typical, with frequent dissatisfaction and distress expressed by both families and medical providers caring for these patients, many of whom were experiencing acute symptoms sometimes requiring inpatient hospitalization, who had no mental health support while waiting to enter treatment. An FBT-based stepped-care model for adolescents with AN, in which the treatment provided was responsive to the unique needs of the patient and their family, was recently shown to yield encouraging rates of remission [42]. Although our brief and group interventions appeared to be feasible, acceptable, and effective from our clinical perspective, in addition to self-reported reductions in provider burnout, we have not empirically evaluated the direct impact of these services on patients, their families, or their medical providers. Qualitative and quantitative data must be collected to truly assess whether these services have positive impacts, as well as when and for whom they are indicated. While we explicitly state that bridge supports are not “substitutes” for treatment, it is possible that offering these services reduced some patients’ and/or caregivers’ sense of urgency to pursue alternative referrals. Conversely, other patients and caregivers have noted that these visits reinforced the importance of taking action rather than maintaining the status quo while waiting to access formal treatment.

Our observations have limited generalizability, first because our clinic primarily serves adolescents with AN and AAN, with an emphasis on FBT. We have less experience implementing such bridge programming with those who are older or have other ED presentations, such as bulimia nervosa or binge eating disorder. Caregivers of children with ARFID were not included in our FBT Caregiver Workshop Series and Support Groups due to their distinct treatment needs. Furthermore, our outpatient clinic is not currently contracted with Medicaid, so patients served were limited to those with commercial insurance or who self-pay. This contributes to health inequities for families and exacerbates existing provider frustration with the inequity in care by insurance type, particularly because patients with Medicaid insurance receive medical care but not behavioral health care within the EDP. Anecdotally, our program’s medical providers and inpatient psychologists often lamented being unable to refer these patients to mental health treatment, including BPCC and the caregiver groups. It is possible that families with fewer economic resources may have encountered unique barriers to participating in and benefiting from the services we have described (e.g., difficulty securing protected time on a set schedule, childcare, privacy, technology required for telehealth visits), but we were unable to pilot these programs more broadly due to our system’s current limitation in serving patients with public insurance.

While efforts are underway to study our bridge programming more rigorously, our clinical observations suggest that these services fill an important gap in care given high rates of referrals and interest from our patients and their families. Many caregivers shared that without access to these resources, they would have been left to navigate their child’s ED treatment in isolation, often using books and other resources to implement a form of self-help. Groups have supported family connection and encouraged communication about EDs, likely aiding families in being better equipped to externalize the ED and reduce stigma. Given the complexity and acuity of EDs, and the importance of early intervention to prevent a prolonged course of illness, these bridge resources may act as a life vest to keep families afloat until more intensive treatment is available. Indeed, clinical observations indicate that connection to bridge services facilitated progress prior to entering formal outpatient treatment, reducing the number of initial sessions required to “get families going” with FBT and potentially reducing the overall number of treatment sessions required. Being able to offer quick access to brief and targeted services is also responsive to family’s consistent requests for any resources that are available while they wait to access treatment, and consistent with our team’s messaging around the urgency of treatment.

Although developed to address a crisis in access to care, these bridge services have been so well-received that we intend to continue them via telehealth as part of routine care. While we would not expect bridge services alone to be sufficient for most families, they may have the potential to reduce use of longer-term outpatient individual or family services, therefore reducing clinical demands overall. The pandemic has exacerbated many longstanding inequities, but it has also provided an opportunity for healthcare systems to think outside-the-box to improve both treatment access and the quality of care delivered. Our hope is that these programs can provide a springboard for other creative efforts to reduce barriers to care. Within our own program, these efforts intensified our team’s commitment to our core values, including delivering evidence-based, accessible care for all young people with EDs, as well as furthering our sense of cohesion and connection. This was critical in boosting our own feelings of efficacy and fulfillment amid the increased burnout brought on by the intersecting professional and personal challenges of treating EDs, and the pandemic [43]. Leveraging our individual and collective strengths, we were able to sustain ongoing programming while developing and implementing new programs, emerging as an even stronger team and more agile program.

## Abbreviations

FBT: Family-based treatment
ED: Eating disorder
EDP: Eating disorders program
UCSF: University of California, San Francisco
AN: Anorexia nervosa
AAN: Atypical anorexia nervosa
OSFED: Other specified feeding and eating disorder
ED-IFT: Intensive family treatment for eating disorders
BPCC: Brief psychology consultation clinic

## Declarations

### Availability of data and materials

Data sharing is not applicable to this article as no datasets were generated or analyzed during the current study.

## References

[1] Zipfel S, Schmidt U, Giel KE (2022). The hidden burden of eating disorders during the COVID-19 pandemic. Lancet Psychiatry.

[2] Rodgers RF, Lombardo C, Cerolini S, Franko DL, Omori M, Fuller-Tyszkiewicz M (2020). The impact of the COVID-19 pandemic on eating disorder risk and symptoms. Int J Eat Disord.

[3] Termorshuizen JD, Watson HJ, Thornton LM, Borg S, Flatt RE, MacDermod CM (2020). Early impact of COVID-19 on individuals with self-reported eating disorders: a survey of~1000 individuals in the United States and the Netherlands. Int J Eat Disord.

[4] Lin JA, Hartman-Munick SM, Kells MR, Milliren CE, Slater WA, Woods ER (2021). The Impact of the COVID-19 pandemic on the number of adolescents/young adults seeking eating disorder-related care. J Adolesc Health.

[5] Hansen SJ, Stephan A, Menkes DB (2021). The impact of COVID-19 on eating disorder referrals and admissions in Waikato, New Zealand. J Eat Disord.

[6] Haripersad YV, Kannegiesser-Bailey M, Morton K, Skeldon S, Shipton N, Edwards K (2021). Outbreak of anorexia nervosa admissions during the COVID-19 pandemic. Arch Dis Child.

[7] Otto AK, Jary JM, Sturza J, Miller CA, Prohaska N, Bravender T (2021). Medical admissions among adolescents with eating disorders during the COVID-19 pandemic. Pediatrics.

[8] Hart LM, Granillo MT, Jorm AF, Paxton SJ (2011). Unmet need for treatment in the eating disorders: a systematic review of eating disorder specific treatment seeking among community cases. Clin Psychol Rev.

[9] Accurso EC, Buckelew SM, Snowden LR (2021). Youth insured by medicaid with restrictive eating disorders: underrecognized and underresourced. JAMA Pediatr.

[10] Becker AE, Franko DL, Speck A, Herzog DB (2003). Ethnicity and differential access to care for eating disorder symptoms. Int J Eat Disord.

[11] Couturier J, Isserlin L, Norris M, Spettigue W, Brouwers M, Kimber M (2020). Canadian practice guidelines for the treatment of children and adolescents with eating disorders. J Eat Disord.

[12] Lebow J, O’Brien JRG, Mattke A, Narr C, Geske J, Billings M (2021). A primary care modification of family-based treatment for adolescent restrictive eating disorders. Eat Disord.

[13] Couturier J, Pellegrini D, Miller C, Bhatnagar N, Boachie A, Bourret K (2021). The COVID-19 pandemic and eating disorders in children, adolescents, and emerging adults: virtual care recommendations from the Canadian consensus panel during COVID-19 and beyond. J Eat Disord.

[14] Fichter MM, Quadflieg N, Crosby RD, Koch S (2017). Long-term outcome of anorexia nervosa: results from a large clinical longitudinal study: Fichter et al.. Int J Eat Disord.

[15] Ágh T, Kovács G, Supina D, Pawaskar M, Herman BK, Vokó Z (2016). A systematic review of the health-related quality of life and economic burdens of anorexia nervosa, bulimia nervosa, and binge eating disorder. Eat Weight Disord.

[16] Allen KL, Byrne SM, Oddy WH, Crosby RD (2013). Early onset binge eating and purging eating disorders: course and outcome in a population-based study of adolescents. J Abnorm Child Psychol.

[17] Forman SF, Grodin LF, Graham DA, Sylvester CJ, Rosen DS, Kapphahn CJ (2011). An eleven site national quality improvement evaluation of adolescent medicine-based eating disorder programs: predictors of weight outcomes at one year and risk adjustment analyses. J Adolesc Health.

[18] Reas DL, Williamson DA, Martin CK, Zucker NL (2000). Duration of illness predicts outcome for bulimia nervosa: a long-term follow-up study. Int J Eat Disord.

[19] Touyz S, Le Grange D, Lacey H, Hay P, Smith R, Maguire S (2013). Treating severe and enduring anorexia nervosa: a randomized controlled trial. Psychol Med.

[20] Lock J, Le Grange D (2019). Family-based treatment: Where are we and where should we be going to improve recovery in child and adolescent eating disorders. Int J Eat Disord.

[21] Eisler I, Simic M, Hodsoll J, Asen E, Berelowitz M, Connan F (2016). A pragmatic randomised multi-centre trial of multifamily and single family therapy for adolescent anorexia nervosa. BMC Psychiatry.

[22] Knatz S, Murray SB, Matheson B, Boutelle KN, Rockwell R, Eisler I (2015). A brief, intensive application of multi-family-based treatment for eating disorders. Eat Disord.

[23] Barney A, Buckelew S, Mesheriakova V, Raymond-Flesch M (2020). The COVID-19 pandemic and rapid implementation of adolescent and young adult telemedicine: challenges and opportunities for innovation. J Adolesc Health.

[24] Joshi H, Corcoran-Lozano P (2021). The behavioral health consultant. Pediatr Clin North Am.

[25] Robinson PJ, Strosahl KD (2009). Behavioral health consultation and primary care: lessons learned. J Clin Psychol Med Settings.

[26] Miller-Matero LR, Dubaybo F, Ziadni MS, Feit R, Kvamme R, Eshelman A (2015). Embedding a psychologist into primary care increases access to behavioral health services. J Prim Care Community Health.

[27] Shonkoff JP, Fisher PA (2013). Rethinking evidence-based practice and two-generation programs to create the future of early childhood policy. Dev Psychopathol.

[28] Rhodes P, Baillee A, Brown J, Madden S (2008). Can parent-to-parent consultation improve the effectiveness of the Maudsley model of family-based treatment for anorexia nervosa? A randomized control trial: parent-to-parent consultation for anorexia. J Fam Ther.

[29] Marzola E, Knatz S, Murray SB, Rockwell R, Boutelle K, Eisler I (2015). Short-term intensive family therapy for adolescent eating disorders: 30-month outcome—short-term intensive family therapy. Eur Eat Disord Rev.

[30] Dennhag I, Henje E, Nilsson K (2021). Parental caregiver burden and recovery of adolescent anorexia nervosa after multi-family therapy. Eat Disord.

[31] Binford Hopf RB, Le Grange DL, Moessner M, Bauer S (2013). Internet-based chat support groups for parents in family-based treatment for adolescent eating disorders: a pilot study—ed parent chat. Eur Eat Disord Rev.

[32] Richards IL, Subar A, Touyz S, Rhodes P (2018). Augmentative approaches in family-based treatment for adolescents with restrictive eating disorders: a systematic review: augmentative approaches in FBT. Eur Eat Disord Rev.

[33] Baudinet J, Eisler I, Dawson L, Simic M, Schmidt U (2021). Multi-family therapy for eating disorders: a systematic scoping review of the quantitative and qualitative findings. Int J Eat Disord.

[34] Ganci M, Pradel M, Hughes EK (2018). Feasibility of a parent education and skills workshop for improving response to family-based treatment of adolescent anorexia nervosa. Int J Eat Disord.

[35] Nicholls DE, Yi I (2012). Early intervention in eating disorders: a parent group approach: EI in eating disorders. Early Interv Psychiatry.

[36] Rosello R, Gledhill J, Yi I, Watkins B, Harvey L, Hosking A (2021). Early intervention in child and adolescent eating disorders: the role of a parenting group. Eur Eat Disord Rev.

[37] Philipp J, Franta C, Zeiler M, Truttmann S, Wittek T, Imgart H (2021). Does a skills intervention for parents have a positive impact on adolescents’ anorexia nervosa outcome? Answers from a quasi-randomised feasibility trial of SUCCEAT. IJERPH.

[38] Zucker NL, Marcus M, Bulik C (2006). A group parent-training program: A novel approach for eating disorder management. Eat Weight Disord.

[39] Le Grange D, Hughes EK, Court A, Yeo M, Crosby RD, Sawyer SM (2016). Randomized clinical trial of parent-focused treatment and family-based treatment for adolescent anorexia nervosa. J Am Acad Child Adolesc Psychiatry.

[40] Lock J, Le Grange D. Treatment manual for anorexia nervosa: a family-based approach. 2., ed.paperback ed. New York: Guilford; 2015. 289 p.

[41] Byrne CE, Accurso EC, Arnow KD, Lock J, Le Grange D (2015). An exploratory examination of patient and parental self-efficacy as predictors of weight gain in adolescents with anorexia nervosa: exploratory examination. Int J Eat Disord.

[42] Le Grange D, Pradel M, Pogos D, Yeo M, Hughes EK, Tompson A (2021). Family-based treatment for adolescent anorexia nervosa: outcomes of a stepped-care model. Int J Eat Disord.

[43] Couturier J, Ma Z, Rahman L, Webb C (2021). A mixed methods exploratory evaluation of burnout in frontline staff implementing dialectical behavior therapy on a pediatric eating disorders unit. J Eat Disord.

